# *lgl* Regulates the Hippo Pathway Independently of Fat/Dachs, Kibra/Expanded/Merlin and dRASSF/dSTRIPAK

**DOI:** 10.3390/cancers6020879

**Published:** 2014-04-16

**Authors:** Linda M. Parsons, Nicola A. Grzeschik, Helena E. Richardson

**Affiliations:** 1 Cell Cycle and Development Laboratory, Peter MacCallum Cancer Centre, Melbourne, Victoria 3002, Australia; E-Mail: helena.richardson@petermac.org; 2 Department of Genetics, University of Melbourne, Melbourne, Victoria 3010, Australia; 3 Sir Peter MacCallum Department of Oncology, University of Melbourne, Melbourne, Victoria 3010, Australia; 4 Department of Anatomy and Neuroscience, University of Melbourne, Melbourne, Victoria 3010, Australia; 5 Department of Biochemistry and Molecular Biology, University of Melbourne, Melbourne, Victoria 3010, Australia

**Keywords:** tumor suppressor, cell polarity, *Drosophila*, Lgl, aPKC, dRASSF, Hpo, Ras, dSTRIPAK complex

## Abstract

In both *Drosophila* and mammalian systems, the Hippo (Hpo) signalling pathway controls tissue growth by inhibiting cell proliferation and promoting apoptosis. The core pathway consists of a protein kinase Hpo (MST1/2 in mammals) that is regulated by a number of upstream inputs including *Drosophila* Ras Association Factor, dRASSF. We have previously shown in the developing *Drosophila* eye epithelium that loss of the apico-basal cell polarity regulator *lethal-(2)-giant-larvae* (*lgl)*, and the concomitant increase in aPKC activity, results in ectopic proliferation and suppression of developmental cell death by blocking Hpo pathway signalling. Here, we further explore how Lgl/aPKC interacts with the Hpo pathway. Deregulation of the Hpo pathway by Lgl depletion is associated with the mislocalization of Hpo and dRASSF. We demonstrate that Lgl/aPKC regulate the Hpo pathway independently of upstream inputs from Fat/Dachs and the Kibra/Expanded/Merlin complex. We show depletion of Lgl also results in accumulation and mislocalization of components of the dSTRIPAK complex, a major phosphatase complex that directly binds to dRASSF and represses Hpo activity. However, depleting dSTRIPAK components, or removal of dRASSF did not rescue the *lgl^−/−^* or aPKC overexpression phenotypes. Thus, Lgl/aPKC regulate Hpo activity by a novel mechanism, independently of dRASSF and dSTRIPAK. Surprisingly, removal of *dRASSF* in tissue with increased aPKC activity results in mild tissue overgrowth, indicating that in this context *dRASSF* acts as a tumor suppressor. This effect was independent of the Hpo and Ras Mitogen Activated Protein Kinase (MAPK) pathways, suggesting that dRASSF regulates a novel pathway to control tissue growth.

## 1. Introduction

The Hpo pathway is conserved from flies to humans and plays a central role in development and disease. Hpo signalling controls organ size by coordinately promoting apoptosis and blocking cell proliferation (reviewed [1]). The core of the Hpo pathway is composed of two kinases: Hpo (orthologue of mammalian sterile 20-like kinase-1 and -2, MST1 and MST2) and Warts (Wts) (orthologue of human large tumor suppressor, LATS1 and LATS2). Hpo and Wts are found in a complex with the scaffolding proteins Salvador (Sav, an orthologue of human WW45) and Mob as tumor suppressor (MATS). Through various upstream pathways, such as the atypical cadherin Fat and/or the FERM domain-containing proteins Expanded (Ex) and Merlin (Mer) in association with Kibra, Hpo senses membrane associated signals and activates Wts through phosphorylation. Upon activation, Wts phosphorylates and inhibits Yorkie (Yki, an orthologue of human Yes-Associated Protein, YAP), a transcriptional coactivator that binds to transcription factors such as Scalloped (orthologue of human Tea Domain proteins, TEAD1-4) and activates expression of cell survival (e.g., *diap1*, *Drosophila inhibitor of apoptosis-1*) and cell proliferation (e.g., *cyclin E*) genes, as well as feedback pathway targets, *expanded* (*ex*) and *four-jointed* (*fj*).

The mammalian Ras Association domain family (RASSF) contains ten different members that fall into two groups, classical (RASSF1-6) and N-terminal (RASSF7-10) proteins (reviewed [2,3]). The defining feature of all RASSF proteins is a Ras association domain (RA) that can be found C-terminally (RASSF1-6) or N-terminally (RASSF7-10). RASSF proteins 1-6 also encode a SARAH protein-protein interaction domain (also present in Salvador, RASSF, Hippo). In *Drosophila*, there is a single, classical *RASSF* gene, *dRASSF*. Similar to its mammalian homologues (RASSF1-6) *dRASSF* encodes a C-terminal Ras Association and SARAH domain. In dRASSF, the SARAH domain mediates dimerization with Hpo and regulates tissue growth via the Hpo tumor suppressor pathway [4].

dRASSF competes with Sav for Hpo binding via the SARAH domain. In doing so, dRASSF inactivates the Hpo kinase cascade, thereby increasing Yki activity and promoting tissue growth [4]. Recent studies in *Drosophila* have identified a phosphatase complex, dSTRIPAK (*Drosophila* Striatin-interacting phophatase and kinase) that directly binds dRASSF, dephosphorylates Hpo and restricts the activity of the pathway to promote tissue growth [5]. Thus, Hpo activity is regulated by a variety of inputs from the cell membrane, and more directly via association with dRASSF and the dSTRIPAK complex.

It has long been recognized that loss-of-function mutations in the *Drosophila* apico-basal cell polarity gene *lethal-giant-larvae (lgl)* result in tissue overgrowth and neoplastic tumors [6]. We have shown in the developing *Drosophila* eye that loss of *lgl* and the concomitant increase in aPKC activity results in ectopic cell proliferation and suppression of developmental cell death (apoptosis) via impairment of the Hpo pathway, as assessed by decreased phospho-Yki and increased expression of target genes (*cycE Diap1*, *ex* and *fj*). Furthermore, when Yki levels were reduced in *lgl^−/−^* tissue ectopic expression of Hpo pathway targets were normalized and overgrowth was decreased, showing that Yki activation was rate-limiting for the tissue overgrowth phenotype conferred by Lgl depletion [7]. We also observed that Lgl/aPKC activity regulates the localization of Hpo/dRASSF [7]. Here we further dissect the relationship between Lgl/aPKC and Hpo pathway regulation. We show that Lgl/aPKC regulate the Hpo pathway independently of upstream components Fat and Kibra/Ex/Mer, the dSTRIPAK complex and dRASSF. Furthermore, *dRASSF* in the context of increased aPKC activity appears to have a tumor suppressor function.

## 2. Experimental

### 2.1. Fly Culture, Overexpression, and Clonal Analysis

Mitotic eye clones and mosaic analysis with a repressible marker (MARCM) clones were generated as previously described [8]. Clonal and MARCM crosses were raised at 25 °C. Overexpression crosses with *GMR* > *aPKC^CAAXWT^* were undertaken at 18 °C.

### 2.2. Fly Stocks

*fat^fd^/CyO* (Kieran Harvey, Peter MacCallum Cancer Centre, Melbourne, Australia); *GMR-GAL4* (Bloomington Stock Centre, Bloomington, IN, USA); *UAS-aPKC^CAAXWT^* (Sonsoles Campuzano, Centro de Biología Molecular Severo Ochoa, Madrid, Spain); *hpo^5.1^/CyO*, *dRASSF^X36^/TM6B* (Nicholas Tapon, Cancer Research, London, UK); *FRT82B*, *kibra^4^/TM6B*, *FRT82B*, *kibra^1^/TM6B* (Hugo Stocker, Institute of Molecular Systems Biology, Zurich, Switzerland); *dachs^GC13^*, *FRT40/CyO*, *dachs^1^/CyO* (Kenneth Irvine, The State University of New Jersey, New Brusnwick NJ, USA); *diap1-GFP1.8* (Duojia Pan, Johns Hopkins University School of Medicine, Baltimore, MD, USA); *diap1-lacZ reporter*, *th^ic58^* (Bruce Hay, California Institute of Technology, Pasadena, CA, USA); *actin* < *CD2 < GAL4*, *UAS-GFP* (Bruce Edgar, Fred Hutchinson Cancer Research Center, Seattle, WA, USA); *eyFLP*; *UAS-RFP*, *tubgal4*, *FRT82B*, *tubgal80/TM6B* (Virender Sahota, Peter MacCallum Cancer Centre, Melbourne, Australia) this study; *merlin (mer)-RNAi^14228R−1^* (National Institute of Genetics (NIG), Shizuoka , Japan); *mob4-RNAi^110742^*, *cka-RNAi^106971^*, *CTTNBP2-RNAi^31377^*, *CCM3-RNAi^106841^*, *lgl-RNAi^51247^* (Vienna *Drosophila* RNAi Centre (VDRC), Vienna, Austria).

### 2.3. Immunohistochemistry, Imaging and Antibodies

**Figure 1:**
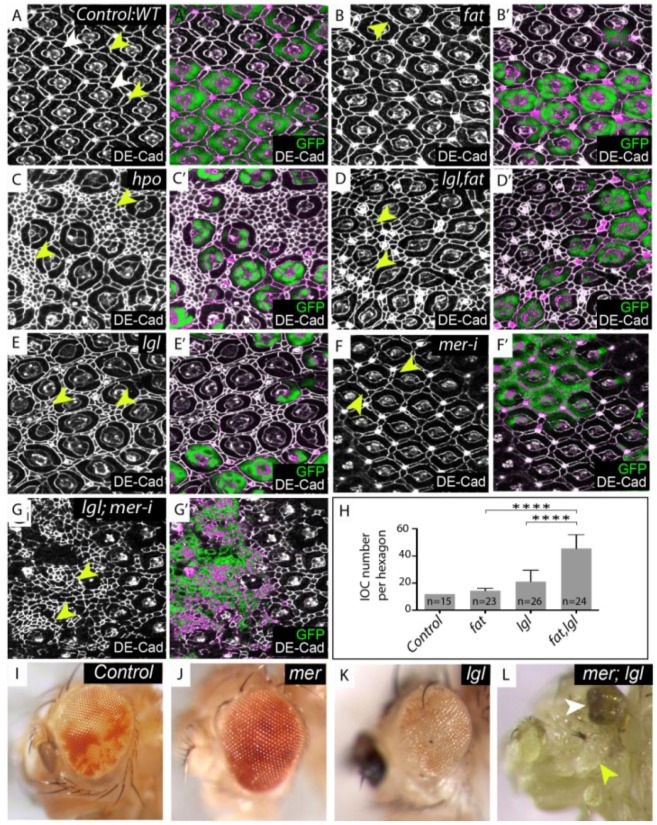
*lgl* regulates the Hippo (Hpo) pathway in parallel with *fat* and *merlin*. (**A**–**G**) Mosaic mid-pupal retinae stained with *Drosophila* E-cadherin (DE-Cad) antibodies that localize to the adherens junction and mark cell outlines. (**A**–**E**) mutant tissue lacks the expression of green fluorescent protein (GFP) and is merged with DE-Cad expression in (**A'**–**E'**). (**A**) Control, wildtype retina containing mosaic tissue expressing GFP and GFP negative tissue, white arrowheads denote photoreceptor and cone cells, yellow arrowheads denote IOCs; (**B**) Retina with *fat*^−/−^ tissue or (**E**) *lgl^−/−^* tissue. The mutant area (GFP negative) shows a few extra IOC’s (yellow arrowheads); (**C**) *hpo^−/−^* clone (GFP negative); (**D**) *lgl*, *fat^−/−^* double mutant tissue (GFP negative) displays a substantial increase in IOC number (yellow arrowheads); (**F**) *mer-RNAi* depleted tissue (marked by the presence of GFP) shows a few additional IOC’s (yellow arrowheads); (**G**) *lgl^−/−^*; *mer-RNAi* tissue (marked by the presence of GFP) display excess IOC’s (yellow arrowheads); (**H**) Mean number of IOCs per ommatidia. **** indicates *p* < 0.0001. Error bars represent Standard Deviation. n equals the number of ommatidia counted; (**I**–**L**) Adult male eye images; (**I**) Control, mosaic eye; (**J**) *mer^−/−^*, mosaic eye (*mer^−/−^* tissue is pale red); (**K**) *lgl^−/−^* mosaic eye (*lgl*^−/−^ tissue is white); (**L**) *mer*; *lgl* double mutant mosaic eye (note the decrease in adult retinal tissue (white arrowhead) and increase in head capsule tissue (yellow arrowhead).

**Figure 2:**
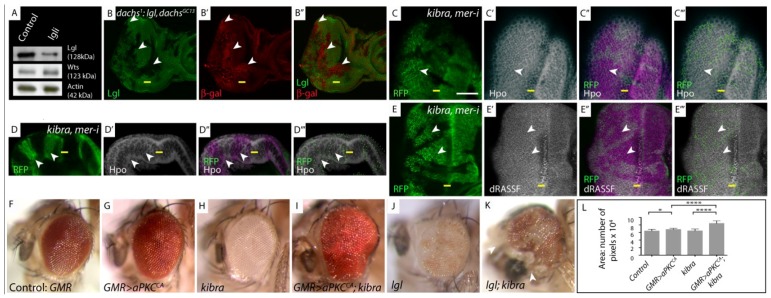
*lgl* regulates the Hpo pathway independently of Fat-Dachs and Kibra/Expanded/Merlin branches. (**A**) Western Blot showing Wts protein levels; (**B**, **C**, **E**) Planar sections of larval eye discs; (**D**) Sideview of larval eye disc. White scale bar represents 50 µM. Yellow bar denotes the morphogenetic furrow (MF), posterior is to the left in planar sections, in this and all other figures; (**F**–**K**) Adult female eye images; (**L**) Graphical representation of adult eye size presented in (**F**–**K**); (**A**) Western Blot analysis of protein extracts derived from control and Lgl depleted larval eye-antennal discs probed for expression of Warts (Wts). There is no change in Wts protein levels in *lgl*-depleted tissue; (**B**) *dachs^1^*: *lgl^−/−^*, *dachs^GC13/GC13^*, *diap1-lacZ* mosaic discs stained for β-gal (red) show increased *diap1-lacZ* staining in *lgl^−/−^*, *dachs^GC13/GC13^* clones (arrowheads). *lgl^−/−^*, *dachs^GC13/GC13^* tissue identified by lack of Lgl antibody staining (green); (**C**,**C'**,**D**,**D'**) Hpo (white) and (**E**,**E'**) dRASSF (white) staining in *kibra*, *mer-RNAi* mosaic eye discs. In *kibra*, *mer-RNAi* tissue (RFP positive, green, mutant tissue indicated by arrowheads) Hpo and dRASSF concentration and localization are normal compared to wildtype tissue (RFP negative); (**C"**–**E"**) Overlay of RFP and antibody staining, pink highlights mutant RFP positive tissue; (**C'''**–**E'''**) Green dots outline RFP clone boundaries; (**F**–**K**) Reducing the level of *kibra* increases the size of *GMR > aPKC^CA^* or *lgl^−/−^* mosaic adult eyes; (**F**) Control adult eye; (**G**) *GMR > aPKC^CA^* adult eye; (**H**) *kibra^−/−^* tissue is white; (**I**) *kibra^−/−^* tissue that expresses *aPKC^CA^* shows synergistic increase in adult eye size; (**J**) *lgl^−/−^* mosaic adult eye (*lgl^−/−^* tissue is white); (**K**) *lgl^−/−^*; *kibra^−/−^* mosaic adult eye (double mutant tissue is white, arrowhead denotes head cuticle tissue); (**L**) Quantification of adult eye size (**F**–**K**).

**Figure 3:**
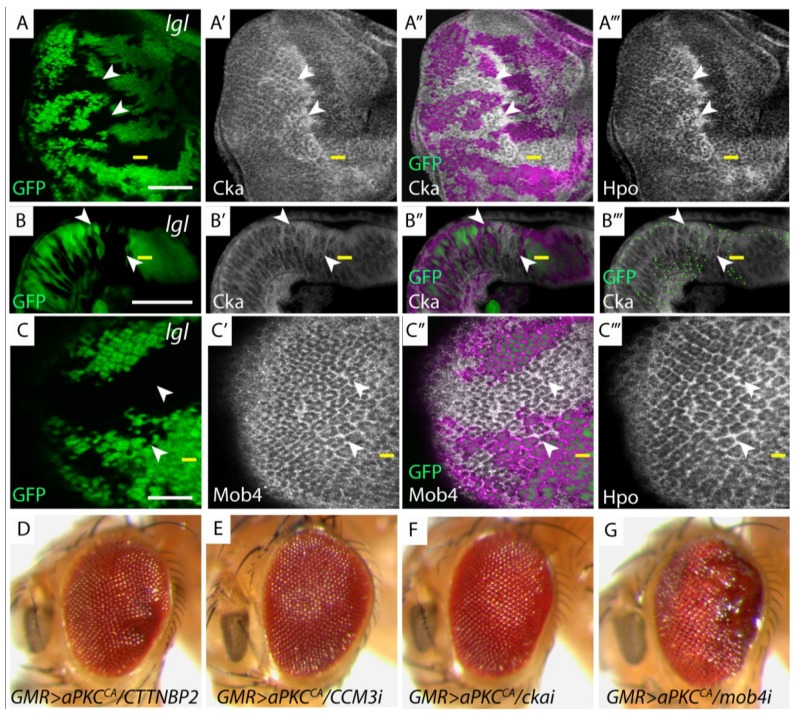
Members of the dSTRIPAK complex are mislocalized in *lgl^−/−^* tissue and genetically interact with *GMR>aPKC^CA^*. (**A**, **C**, **H**, **I**) Planar sections of larval eye discs; (**B**) Cross section of larval eye disc. Yellow line indicates morphogenetic furrow. White scale bar represents 50 µM; (**A**, **A'**, **A'''**) *lgl^−/−^* mosaic eye disc stained for Cka (white) and Hpo (white) respectively. Mutant clones display apical accumulation of Hpo and Cka (arrowheads); (**B**, **B'**) *lgl^−/−^* mosaic eye disc, stained for Cka (white) displays apical accumulation and basolateral mislocalization of Cka (mutant tissue GFP negative, white arrowheads indicate Cka mislocalization); (**B'''**) Green dots outline GFP clone boundaries; (**C**, **C'**, **C'''**) *lgl^−/−^* mosaic eye disc, stained for Mob4 (white) and Hpo (white) respectively. Only moderate accumulation of Mob4 was observed compared to Hpo (mutant tissue GFP negative, arrowheads indicate Mob4 mislocalization); (**A"**–**C"**) Overlay of GFP and antibody staining, pink highlights wildtype GFP positive tissue; (**D**–**G**) Adult female eye images of *GMR > aPKC^CA^* and depletion of dSTRIPAK components; (**D**) *CTTNBP2*; (**E**) *CCM3i*; (**F**) *ckai*; (**G**) *mob4.* Reducing the levels of *mob4*, but not other dSTRIPAK components, in conjunction with *GMR* > *aPKC^CA^* alters tissue growth.

**Figure 4:**
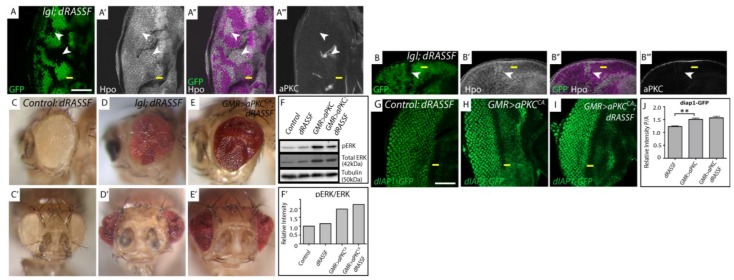
Hpo mislocalization in *lgl^−/−^* mosiac tissue is independent of *dRASSF* and *dRASSF* acts as a tumor suppressor. (**A**, **G**–**I**) Planar sections of larval eye discs; (**B**) Cross section of larval eye disc. Yellow line indicates morphogenetic furrow. White scale bar represents 50 µM. (**C**–**E**) Adult female eye images. (**A**) *lgl*; *dRASSF* double mutant tissue (GFP negative) displays apical accumulation of Hpo (white, arrowhead indicates double mutant, GFP negative tissue) compared to *dRASSF* mutant tissue (GFP positive) and is mislocalized basolaterally in *lgl*; *dRASSF* double mutant tissue (**B** arrowhead). aPKC staining (white) is altered where the tissue shows obvious folds (**A**, white asterik) but is still localized apically *in lgl*; *dRASSF* double mutant tissue (**B**); (**C**, **C'**, **D**, **D'**, **E**, **E'**) Adult female eye images, side and ventral views respectively; (**C**,**C'**) *dRASSF*; (**D**, **D'**) *lgl*; *dRASSF*; (**E**, **E'**) *GMR > aPKC^CA^*, *dRASSF.* The eye size of *lgl* mosaic and *GMR > aPKC^CA^* overexpressing adult eyes is increased when *dRASSF* is removed; (**F**) Western blot of protein extracts from eye discs detecting pERK, total ERK and tubulin levels; (**F'**) Graphical representation of signal intensity from Western blots. There is a two fold increase in pERK/ERK levels in *GMR > aPKC^CA^* mutant tissue. However, removal of *dRASSF* does not alter the relative ratio of pERK/ERK in either a wildtype or *GMR > aPKC^CA^* tissue; (**G**–**H**) *dRASSF*, *GMR > aPKC^CA^*, *GMR > aPKC^CA^*; *dRASSF* crossed with *diap1-GFP1.8.* GFP expression (green) reports *diap1* expression. Compared with the control; (**G**) *dRASSF*; (**H**) *GMR > aPKC^CA^*; and (**I**) *GMR > aPKC^CA^*; *dRASSF* discs show elevated levels of *diap1-GFP1.8* expression but there is no increase in *diap1-GFP1.8* expression between *GMR > aPKC^CA^* and *GMR > aPKC^CA^*; *dRASSF* tissue; (**J**) Graph of the ratio of intensity from posterior *versus* anterior regions of *diap1-GFP1.8* expressing samples, taken from apical section. The upregulation of *diap1-GFP1.8* in *GMR > aPKC^CA^* and *GMR > aPKC^CA^*; *dRASSF* tissue is significant compared to *dRASSF* alone but there is no significant difference between *GMR > aPKC^CA^* and *GMR > aPKC^CA^*; *dRASSF* tissues. *n* = 5 discs per sample. Error bars represent Standard Error of the Mean. ** indicates *p* < 0.001.

**Figure A1:**
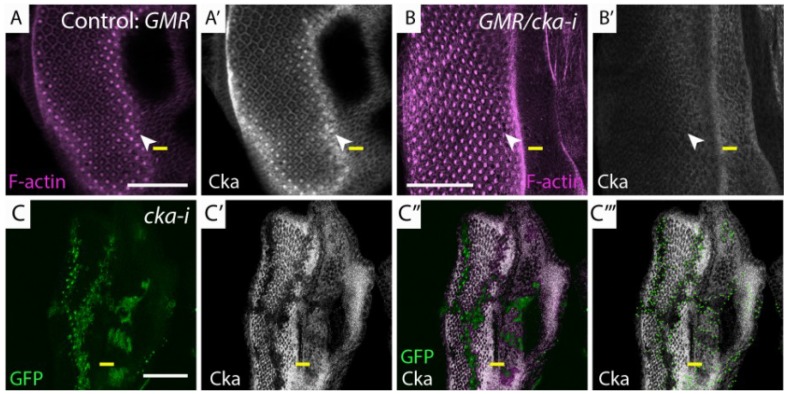
Validation of *cka-i*. (**A**–**C**) Planar sections of larval eye discs. Yellow line indicates morphogenetic furrow. White scale bar represents 50 µM. (**A**,**A'**) Control; *GMR* eye disc stained with F-actin and Cka revealing the apical surface of eye disc; (**B**,**B'**) *GMR/cka-i* eye disc stained with F-actin and Cka, note apical depletion of Cka staining posterior to the morphogenetic furrow; (**C**,**C'**) *cka-i* clones (GFP tissue) show depletion of Cka staining (white); (**C"**,**C'''**) overlay of GFP positive tissue and Cka staining confirms lack of Cka staining in clones expressing *cka-i* (pink), *cka-i* clone boundaries outlined with green dots. (**A**) Control: GMR: *GMR-GAL4/+*; (**B**) *GMR/cka-i*: *GMR-GAL4/UAS-cka-RNAi^1^°^6971^*; (**C**) *cka-i*: *eyFLP*, *UAS-GFP*; *gal80*, *FRT40/UAS-cka-RNAi^1^°^6971^*, *FRT40*; *tubgal4/+*.

Larval and pupal discs were dissected and fixed as previously described [7]. For Hpo, dRASSF, Cka and Mob4 staining, tissues were fixed in paraformaldehyde lysine periodate (PLP). Labeled samples were cleared through 80% glycerol and mounted. For Figure 1, Figure 2E, Figure 3A,C, Appendix Figure A1B,C, and Figure 4A,B images were collected on Biorad MRC1000 (Bio-Rad Laboratories, Hercules, CA, USA). Figure 2B, Figure 3B and Appendix Figure A1A were collected on an Olympus FV1000 (Olympus, Center Valley, PA, USA). Images in Figure 2C,D and Figure 4G–I were taken on a Nikon Eclipse 90i (Nikon, New York, NY, USA). Images were processed using Fiji, and assembled with Adobe Photoshop CS6 and Adobe Illustrator CS6. Adult eyes were imaged with a Scitec Infinity1 camera (Lumenera, Ottawa, Canada).

Antibodies used were mouse anti-β-galactosidase (Sigma, St. Louis, MO, USA, 1:500), rat anti-Hpo (1:100), rabbit anti-dRASSF (1:200) both from Nicholas Tapon, Cancer Research, UK, rabbit anti-Cka (Wei Du, University of Chicago, Chicago, IL, USA 1:1000) rabbit anti-Lgl (Dennis Strand, Johannes Gutenberg University, Germany. 1:1000), guinea-pig anti-Mob4 (Joost Schulte, University of British Columbia, Vancouver, Canada, 1:1000), rat anti-Shg (E-cadherin, Developmental Studies Hybridoma Bank, DSHB, University of Iowa, IA, USA. 1:50), rabbit anti-aPKC (Santa Cruz Biotechnology, Dallas, TX, USA, human PKCζ 1:500).

### 2.4. Quantification of Interommatidial Cell (IOC) Number

The number of IOCs (secondary and tertiary pigment cells) for a single ommatidium was quantified by drawing a hexagon connecting the centers of the six surrounding neighboring ommatidia. IOCs within the hexagon were then counted. IOCs that straddled the boundary were counted as half a cell.

### 2.5. Quantification of Adult Eye Size

The perimeter of adult eyes was outlined in Photoshop CS3 and the total area in pixels calculated. Data was analysed using unpaired *t*-test (Welch corrected).

### 2.6. Statistical Analysis of Signal Intensity

To determine the relative *diap1-GFP1.8* expression in eye discs, images of each disc were taken with identical confocal settings. Using Image J, a ratio of average pixel intensity was determined for *diap1-GFP1.8* immunofluorescence in an area posterior and anterior to the morphogenetic furrow that colocalized with DAPI staining. Data was analyzed using unpaired *t*-test (Welch corrected).

(**A**) Control: *eyFLP*; *FRT40/UbiGFP*, *FRT40*; (**B**) *fat: eyFLP*; *fat^fd^*, *FRT40/UbiGFP*, *FRT40*; (**C**) *hpo*: *eyFLP*, *FRT42D*, * hpo^5.1^/FRT42D*, * UbiGFP*; (**D**) *lgl*, *fat*: *eyFLP*, *lgl^27S3^*, *fat^fd^*, *FRT40/UbiGFP*, *FRT40*; (**E**) *lgl*: *eyFLP*, *lgl^27S3^*, *FRT40/UbiGFP*, *FRT40*; (**F**) *mer-i*: *eyFLP*, *UAS-GFP*; *FRT40/tubgal80*, *FRT40*; *UAS mer-RNAi^14228R^/tubgal4*; (**G**) *lgl*;*mer-i*: *eyFLP*, *UAS-GFP*; *lgl^27S3^*, *FRT40/gal80*, *FRT40*; *UAS-mer-RNAi^14228R^/tubgal4*; (**I**) *Control*: *eyFLP*; *FRT40/UbiGFP FRT40*; (**J**) *mer*: *y w Mer^4^ FRT19A/Y*; *FRT40A/P[w^+^ Mer^+^]*, *Ubi-GFP*, *FRT40A*; *eyGAL4*, *UAS-flp/+*; (**K**) *lgl*: *eyFLP*; *lgl^27S3^*, *FRT40/UbiGFP*, *FRT40*; (**L**) *mer*; *lgl*: *y w Mer^4^ FRT19A/Y*; *P[w^+^ Mer^+^] Ubi-GFP FRT40A/lgl^27S3^ FRT40A*; *eyGAL4*, *UAS-flp/+.*

(**A**) Control: *eyFLP*; *actin < CD2 < GAL4; UAS-GFP*; *lgli*: *eyFLP*; *actin < CD2 < GAL4; UAS-GFP/UAS-lgl-RNAi^51247^*; (**B**) *dachs^1^*: *lgl*, *dachs^GC13^*: *eyFLP*; *lgl^27S3^*, *dachs^GC13^*, *FRT40/dachs^1^*, *FRT40*; *diap1-lacZ reporter (th^ic58^)/+*; (**C**–**E**) *kibra*, *mer-i*: *eyFLP*; *FRT82B*, *kibra^4^*, *UAS-merlin RNAi^14228R−1^/UAS-mRFP*, *tubgal4*, *FRT82B*, *tubgal80*; (**F**) Control: *GMR-GAL4/+*; (**G**) *GMR > aPKC^CA^*: *GMR-GAL4 > UAS-aPKC^CAAXWT^/+*; (**H**) *kibra*: *eyFLP*; *FRT82B*, *kibra^1^/FRT82B*, *P{w+} cell lethal*; (**I**) *GMR > aPKC^CA^*; *kibra*: *eyFLP*; *GMR-GAL4 > UAS-aPKC^CAAXWT^/+*; *FRT82B*, *kibra^1^/FRT82B*, *P{w+} cell lethal*; (**J**) *lgl*: *eyFLP*; *lgl^27S3^*, *FRT40/Ubi-GFP*, *FRT40*; (**K**) *lgl*, *kibra*: *eyFLP*; *lgl*^27S3^, *FRT40/Ubi-GFP*, *FRT40*; *FRT82B*, *kibra^1^/FRT82B*, *P{w+} cell lethal*; (**L**) Mean eye size represented by the area * indicates *p* < 0.05; **** indicates *p* < 0.0001. Error bars represent Standard Deviation. n = 12 eyes per genotype.

(**A**–**C**) *lgl*: *eyFLP*; *lgl^27S3^*, *FRT40/UbiGFP*, *FRT40*; (**D**) *GMR > aPKC^CA^/CTTNBP2*: *GMR-GAL4 > UAS-aPKC^CAAXWT^/UAS-CTTNBP2-RNAi^31377^*; (**E**) *GMR > aPKC^CA^/CCM3i*: *GMR-GAL4 > UAS-aPKC^CAAXWT^/UAS-CCM3-RNAi^106841^*; (**F**) *GMR > aPKC^CA^/ckai*: *GMR-GAL4 > UAS-aPKC^CAAXWT^/UAS-cka-RNAi^106971^*; (**G**) *GMR > aPKC^CA^/mob4i*: *GMR-GAL4>UAS-aPKC^CAAXWT^/UAS-mob4-RNAi^11074^.*

(**A**,**B**) *lgl*; *dRASSF*: *eyFLP*; *lgl^27S3^*, *FRT40/UbiGFP*, *FRT40*; *dRASSF^X36/X36^*; (**C**) *Control dRASSF*: *dRASSF^X36/X36^*; (**D**) *lgl*; *dRASSF*: *eyFLP*; *lgl^27S3^*, *FRT40/UbiGFP*, *FRT40*; *dRASSF^X36/X36^*; (**E**) *GMR>aPKC^CA^*; *dRASSF*: *GMR-GAL4 > UAS-aPKC^CAAXWT^*; *dRASSF^X36/X36^*; (**F**) *Control*: *w1118*, *dRASSF*: *dRASSF^X36/X36^*, *GMR>aPKC^CA^*: *GMR-GAL4 > UAS-aPKC^CAAXWT^ GMR>aPKC^CA^*; *dRASSF*, *GMR-GAL4 > UAS-aPKC^CAAXWT^*; *dRASSF^X36/X36^*; (**G**) *Control dRASSF*: *GMR-GAL4 > diap1-GFP1.8/+*; *dRASSF ^X36/X36^*; (**H**) *GMR > aPKC^CA^*: *GMR-GAL4 > diap1-GFP1.8/UAS-aPKC^CAAXWT^*; (**I**) *GMR>aPKC^CA^*; *dRASSF*: *GMR-GAL4 > diap1-GFP1.8/UAS-aPKC^CAAXWT^*; *dRASSF ^X36/X36^.*

### 2.7. Sample Preparation and Western Blot Analysis

Eye-antennal discs were dissected from *w1118*, *dRASSF^X36/X36^*, *GMR > aPKC^CAAXWT^*, *GMR > aPKC^CAAXWT^*; *dRASSF^X36/X36^* or *ey-FLP* and *eyFLP*; *lgli actin-FLP*-out larvae, homogenized in 0.1 M Tris-HCl pH 6.8, 2% SDS. 5 mM EDTA, 5 mM DTT buffer containing Complete protease inhibitor cocktail (Roche, Basel, Switzerland), 1 mM Na_3_(VO)_4_, 5 mM NaF_2_. In Figure 2A, protein concentration was determined by Lowry assays. Samples containing 20 mg of protein were electrophoresed on a 10% SDS-PAGE gel and transferred to Immobilon-FL polyvinylidene difluoride membranes. In Figure 4F, 11 eye disc pairs were dissected per sample and electrophoresed as above. Antibodies used in Figure 2A were rabbit anti-Lgl (Dennis Strand, Johannes Gutenberg University, Germany. 1:1000), rabbit anti-Wts (Kenneth Irvine, The State University of New Jersey, NJ, USA. 1:500) and mouse anti-actin (Developmental Studies Hybridoma Bank, DSHB, University of Iowa, IA, USA. 1:100) and in Figure 4F were mouse anti-pERK (Sigma, St. Louis, MO, USA., 1:10,000), anti rabbit-ERK 137 F5 (Cell Signalling, Danvers, MA, USA 1:1000) and mouse anti-α-tubulin (Calbiochem, MERCK, Darmstadt, Germany 1:10,000). The signal intensity of bands in Figure 2A and Figure 4F was measured in Fiji and relative pERK/ERK levels were normalized to α-tubulin.

## 3. Results

### 3.1. lgl Regulates the Hpo Pathway Independently of Fat and Dachs

To determine how Lgl regulates the Hpo pathway we asked if loss of *lgl* function affected the phenotypes of known upstream regulators of the Hpo pathway. First, we assessed if Lgl genetically interacted with the atypical cadherin Fat [9,10,11]. A defining characteristic of Hpo pathway mutants is an increase in cell number in the pupal retina [12]. The *Drosophila* eye develops from the eye imaginal disc, a single-cell-layered epithelial sheet that grows during larval stages. After photoreceptor and cone cells are determined, interommatidial cells (IOC’s) are specified to become pigment cells, with excess IOC’s are eliminated by apoptosis. Interommatidial (or pigment cells) surround the centrally located photoreceptor and cone cells, generating a precise, repeating hexagonal structure (ommatidium), which can be visualized during the pupal stage (Figure 1A; white arrowheads mark photoreceptor, cone and primary pigment cells, yellow arrowheads indicate secondary and tertiary pigment cells). In mutants such as *hpo^−/−^*, where developmental cell death is inhibited, IOC’s accumulate between the ommatidia of the pupal retina (Figure 1C; yellow arrowhead). *lgl^−/−^* tissue also fails to undergo programmed cell death and a few extra cells can be observed in *lgl^−/−^* retinae (Figure 1E; yellow arrowhead), however this phenotype is not as strong as that displayed by *hpo^−/−^* tissue (compare Figure 1C,E). In contrast to *hpo^−/−^* retinae, *fat^−/−^* tissue showed only a few extra cells (Figure 1B yellow arrowhead). Notably, *lgl*, *fat* double mutant tissue showed a greater number of IOC’s than either *lgl* or *fat* single mutant retinae (compare Figure 1D with Figure 1B,E). Quantification of the number of IOC’s per ommatidia in *fat* or *lgl* single mutants revealed an average of two or eight additional cells respectively. Strikingly the average number of IOC’s per ommatidia in *lgl*, *fat* double mutant tissue was thritythree (quantified in Figure 1H). This data shows that *lgl* and *fat* act synergistically to regulate IOC number during eye development, and therefore might function redundantly within the same pathway or in parallel pathways.

We then wished to determine if Lgl and Fat acted together within the same complex or independently of each other to regulate Hpo signalling. Therefore we analyzed if Wts protein levels were reduced in *lgl^−/−^* tissue. Fat regulates the Hpo pathway, in part by regulating the stability of Wts kinase. Wts protein levels are reduced in *fat^−/−^* tissue and this decrease in Wts is dependent on the activity of an unconventional myosin, Dachs [13,14]. In *fat*, *dachs* double mutant tissue, the loss of *dachs* restores Wts protein levels, thereby increasing the levels of phospho-Yki and downregulating target gene expression [13].

We previously used the *ey-FLP-actin-FLP-out* system combined with a *lgl-RNAi* transgene to specifically knockdown Lgl and showed that when Lgl is depleted in the eye-antennal imaginal disc phospho-Yki levels decrease and Yki target gene expression is increased [7]. When Lgl was knocked down by 80% using *ey-FLP-actin-FLP-out* (Figure 2A), Western blot analysis showed that there was no statistically significant change (unpaired t-test Welch’s correction, *p* > 0.05, n = 4) in Wts protein levels between control eye-antennal tissue and *lgl-RNAi* depleted tissue (Figure 2A). This data shows that *lgl* does not mediate Hpo pathway activation by decreasing Wts protein levels.

We have shown that *lgl^−/−^* tissue upregulated the *diap1* transcriptional reporter *diap1-lacZ* [7]. To test the requirement of *dachs* in this phenotype, we investigated whether removing *dachs* would reduce the elevated level of *diap1-lacZ* expression in *lgl^−/−^* tissue to wildtype. *dachs^GC13/GC13^* clones grow poorly when surrounded by wildtype tissue [14], therefore we generated *lgl*, *dachs^GC13/GC13^* clones in a *dachs^1^* background. This enabled us to recover *lgl*, *dachs^GC13/GC13^* clones in larval eye discs, however *lgl*, *dachs^GC13/GC13^* clones were not observed in adult eyes (data not shown). We found that in *lgl*, *dachs^GC13/GC13^* tissue upregulation of *diap1-lacZ* persisted (arrowheads Figure 2B–B'''). Thus, activation of Yki target genes upon depletion of Lgl is independent of *dachs* function.

Taken together, the synergistic effects on increased cell number observed in *lgl*, *fat* double mutant pupal retinas, maintenance of elevated *diap1-lacZ* gene expression in *lgl*, *dachs* double mutant tissue, and the lack of change in Wts protein levels in *lgl-RNAi* depleted eye-antennal discs, strongly supports the notion that *lgl* acts separately and in parallel with Fat/Dachs to regulate the Hpo pathway.

### 3.2. lgl Regulates the Hpo Pathway Independently of the Apical Kibra/Expanded/Merlin Complex

The discovery of the apical FERM domain containing complex Kibra/Expanded/Merlin (KEM) as a regulator of Hpo signalling [15,16,17], and that in mammalian systems, Kibra has been shown to be a direct target of aPKC phosphorylation [18], raised the possibility that *lgl* may regulate Hpo pathway activity via the KEM complex.

Initially we investigated tissue growth phenotypes of *lgl^−/−^*; *ex-RNAi* or *lgl^−/−^*; *mer-RNAi* depleted larval eye discs. *lgl^−/−^*; *ex-RNAi* or *lgl^−/−^*; *mer-RNAi* depleted larval eye discs showed excess tissue growth, greater than single mutants alone, and these animals mostly failed to pupate (data not shown). In one *lgl^−/−^*, *mer-RNAi* depleted pupal disc that we were able to recover, we observed a significant increase in IOC’s, (compare Figure 1E–G), similar to that observed for *lgl*, *f*at double mutants (compare Figure 1D,G). Consistent with the breakdown of tissue observed in the *lgl^−/−^*;*mer-RNAi* pupal retina (Figure 1G), rare adult male escapers double mutant for *mer* and *lgl* showed a dramatic loss of differentiated adult eye tissue (white arrowhead) and an increase in head capsule tissue (yellow arrowhead, compare Figure 1I–L). The increase in IOC’s in *lgl^−/−^*; *mer-RNAi* retina and increased tissue growth of *lgl^−/−^*; *ex-RNAi* larval eye discs shows that Lgl and the KEM complex act synergistically which might indicate that they act redundantly in the same pathway or in parallel pathways to regulate the Hpo pathway.

In our analysis of the effect of Lgl depletion/aPKC activation on the localization of Hpo pathway components, we observed co-mislocalization of Hpo and dRASSF, and proposed that Lgl/aPKC regulate the Hpo pathway through Hpo/dRASSF apical localization [7]. To determine if components of the KEM complex and Lgl regulate the Hpo pathway via redundant or independent mechanisms we investigated whether the localization of Hpo and dRASSF was altered by depletion of the KEM complex. As members of the KEM complex are functionally redundant with one another we chose to undertake these studies in a stronger double mutant background of *kibra^−/−^*, *mer-RNAi* (the efficacy of the *mer-RNAi* is demonstrated in Figure 1G and discussed above). We have previously shown that Hpo and dRASSF proteins accumulate apically and are mislocalized basolaterally in *lgl*^−/−^ tissue [7], (demonstrated here again in Figure 3A,C, showing apical accumulation of Hpo in *lgl^−/−^* tissue). In contrast, in *kibra^−/−^*, mer-RNAi tissue, Hpo and dRASSF showed normal apical levels (Figure 2C,D, RFP positive tissue, arrowheads). Furthermore, a sideview of a *kibra^−/−^*, *mer-RNAi* eye disc (Figure 2D) showed no basolateral mislocalization of Hpo. Thus, *kibra* and *merlin* activity do not regulate the localization of Hpo and dRASSF.

To further investigate the genetic interaction between *lgl* and *kibra*, we generated adult eyes comprised entirely of *kibra* mutant tissue and asked what happened to tissue growth if we generated *lgl* mutant tissue, or overexpressed a constitutively membrane-bound (active) version of aPKC (*aPKC^CAAXWT^* hereafter referred to as *aPKC^CA^*) in this genetic background. *lgl^−/−^*; *kibra^−/−^* adult eyes have increased tissue growth compared to *kibra^−/−^* or *lgl^−/−^* alone (compare Figure 2H,J,K). This is particularly noticeable in head capsule tissue that shows excessive tissue growth (Figure 2K, arrowheads). As previously reported [17], adult eyes comprised of entirely *kibra^−/−^* tissue showed a weak increase in eye size (3%) when compared with control eyes (compare Figure 2F,H, quantified in Figure 2L). *GMR > aPKC^CA^* adult eyes showed a 12% increase in eye size compared with control eyes (compare Figure 2F,G, quantified in Figure 2L). Consistent with the phenotype of adult eyes double mutant for *lgl* and *kibra* (Figure 2K), adult eyes mutant for *kibra* and overexpressing *aPKC^CA^* (*GMR > aPKC^CA^)* have a synergistic increase in eye size (38% compared to control eyes), greater than *kibra^−/−^* or *GMR > aPKC^CA^* alone (Figure 2I, quantified in Figure 2L). Taken together, the normal levels and localization of Hpo and dRASSF in *kibra^−/−^*, *mer-RNAi* tissue, and genetic interaction data showing that *kibra^−/−^* and *mer-RNAi* act synergistically with *lgl^−^*^/−^ or *aPKC^CA^* overexpression to regulate tissue growth, supports the notion that Lgl/aPKC regulate the Hpo pathway independently of the apical KEM complex.

### 3.3. Members of the dSTRIPAK Complex Are Mislocalized in lgl Mutant Tissue

The discovery of a PP2A phosphatase complex, dSTRIPAK, which exclusively interacts with dRASSF/Hpo and not Hpo/Sav [5], raised the possibility that Lgl/aPKC regulates the Hpo pathway by modulating dSTRIPAK activity. In human cells, PP2A complex formation is achieved through the combinatorial assembly of two PP2A catalytic subunits, a regulatory B subunit and two scaffolding subunits (reviewed [19]). The dSTRIPAK complex associated with Hpo also contains two catalytic subunits, a regulatory subunit Cka (Connector of kinase to AP-1) and a kinase Mob4 (Mps one binder kinase activator-like 4). We used immunohistochemistry to determine, if similar to Hpo and dRASSF, Cka and Mob4 are mislocalized in *lgl^−/−^* tissue. In the developing *Drosophila* larval eye disc, Hpo, Cka and Mob4 localized apically and associated with cell membranes to outline the developing ommatidial clusters (Figure 3A,C). Like Hpo, in *lgl^−/−^* tissue, Cka accumulated apically compared with the surrounding wildtype tissue (Figure 3A arrowheads). Cross section of the eye disc revealed that in *lgl^−/−^* clones posterior to the morphogenetic furrow there was apical accumulation and basolateral mislocalization of Cka (Figure 3B, *lgl^−/−^* tissue denoted by arrowheads). The change in Cka localization in *lgl^−/−^* tissue is independent of changes in apico-basal cell polarity, because we have previously shown that cell polarity is maintained in *lgl^−/−^* larval eye disc clones [7]. Mob4 and Hpo staining partially overlapped in *lgl^−/−^* tissue. Although there were some regions of the eye disc that showed some accumulation of Mob4 (Figure 3C, arrowhead) Mob4 was less affected than Hpo (Figure 3C arrowhead). Taken together, these results show that Lgl is required for the correct localization of dRASSF and Hpo, and members of the phosphatase dSTRIPAK complex, Cka and Mob4.

We predicted that mislocalization of negative regulators of the Hpo pathway such as dRASSF and members of the dSTRIPAK complex, lead to the observed decrease in Hpo pathway activity and the subsequent upregulation of Yki pathway targets in *lgl^−/−^* tissue. Therefore, reduction of negative regulators of the Hpo pathway, such as dSTRIPAK [5], might restore Hpo signalling and reduce tissue growth. We used the *GMR > aPKC^CA^* phenotype, which shows mislocalization of Hpo proteins, upregulation of Hpo pathway targets and a weak adult eye overgrowth phenotype (Figure 2G, quantified 2L), to determine if depletion of dSTRIPAK complex members could suppress aPKC mediated Hpo pathway activity. To achieve this, we depleted each dSTRIPAK member (*CCM3 (CG5073)*, *SLMAP (CG17494)*, *CTTNBP2 (CG10915)*, *FAM40A (CG11526)*, *FGOP2 (CG10158)*, *cka* and *mob4*) using RNAi in the developing *Drosophila* eye via the *GMR* driver. Knockdown of dSTRIPAK members alone via the *GMR* driver produced no observable effects. However, as predicted [5], all RNAi’s enhanced the *GMR-GAL4*, *UAS-hpo* phenotype at 18 °C and were late pupal lethal displaying severe eye and head defects (data not shown). Moreover, examination of *GMR-GAL4* induced knockdown of Cka at 18 °C, or in clones at 18 °C, revealed a significant depletion of apically-localized Cka protein (compare Cka staining in control disc to *cka*-*RNAi* depleted disc Supplementary Figure 1A,B, arrowheads and in clones 1C). However, when tested for their interaction with *GMR > aPKC^CA^*, none of the dSTRIPAK complex component depletions suppressed the *GMR > aPKC^CA^* phenotype (Figure 3D–F; and data not shown). Conversely, *mob4* depletion in conjunction with *GMR > aPKC^CA^*, resulted in a slight increase in adult eye size and an accumulation of glassy tissue in the posterior region of the adult eye (Figure 3G) relative to *GMR > aPKC^CA^* alone. These data show that only one dSTRIPAK complex member, Mob4, showed a genetic interaction with Lgl/aPKC. However, this interaction is opposite to what would be expected if dSTRIPAK is responsible for the decreased Hpo activity in *aPKC^CA^* over-expressing tissue. Thus, depletion of the dSTRIPAK complex components does not reduce tissue growth in *GMR > aPKC^CA^* adult eye, and therefore the dSTRIPAK complex is not a critical factor in the impairment of Hpo activity by activated aPKC.

### 3.4. lgl Mutant Tissue Mislocalizes and Inactivates Hpo Pathway Signalling Independently of dRASSF Function

Previous studies have shown that Hpo and dRASSF form a protein complex that antagonizes Hpo pathway activity [3]. We wished to determine if *dRASSF* was required to mislocalize Hpo proteins in *lgl^−/−^* tissue. To achieve this, we generated *dRASSF* homozygous mutant animals that also contained mosaic *lgl^−/−^* tissue in the developing eye and examined Hpo localization. Hpo proteins still accumulated apically in *lgl^−/−^*; *dRASSF^−/−^* tissue (Figure 4A arrowheads) and in cross sections of the developing eye disc, Hpo was clearly mislocalized basolaterally (arrowheads Figure 4B). The basolateral changes were not associated with cell polarity changes in *lgl^−/−^* tissue because aPKC localization remains unchanged (Figure 4B). These data show that although Hpo and dRASSF directly bind to each other, this interaction is not required for the localization of Hpo in either *dRASSF* single mutant, or *lgl*; *dRASSF* double mutant tissue.

### 3.5. dRASSF Acts as a Tumor Suppressor in lgl Mutant and GMR>aPKC^CA^ Tissue

As stated previously, we predicted that mislocalization of negative regulators of the Hpo pathway, such as dRASSF, led to the observed upregulation of Hpo pathway targets in *lgl^−/−^* tissue. Therefore, reduction in *dRASSF* activity might restore Hpo signalling and reduce tissue growth in *lgl^−/−^* or *GMR>aPKC^CA^* tissue. To achieve this, we investigated the genetic interaction between *lgl^−/−^*, *GMR>aPKC^CA^* and *dRASSF*. Adult eyes from homozygous *dRASSF* single mutants develop and differentiate normally, but showed slightly reduced adult eye size consistent with an overall decrease in body size [4], (Figure 4C,C'). When *dRASSF* activity was removed in *lgl^−/−^* mosaic or *GMR > aPKC^CA^* animals the resulting adult eyes were larger and showed tissue folds, consistent with increased tissue growth (Figure 4D,D',E,E' respectively). These data were opposite to that expected if *dRASSF* was acting to restrict Hpo function in *lgl^−/−^* or *GMR > aPKC^CA^* eyes, and instead demonstrate that *dRASSF* is required to restrict growth when aPKC activity is increased. These data suggest that *dRASSF* may act a tumor suppressor in this context.

We predicted that loss of *dRASSF* would restore Hpo pathway signalling and decrease Yki target gene expression in *GMR > aPKC^CA^* tissue. Therefore, we determined if Hpo pathway activation was suppressed and Yki target gene expression decreased by determining the levels of *diap1-GFP* reporter gene expression, (*diap1-GFP1.8*, which contains enhancer regions critical for the activation of *diap1* expression by Yki [20]). *GMR > aPKC^CA^* and *GMR > aPKC^CA^*; *dRASSF^−/−^* eye discs show upregulation of *diap1-GFP1.8* reporter gene expression (compare Figure 4G–J) when compared to control *dRASSF* single mutant eye discs (quantified in Figure 4J) but there was no statistically significant change of *diap1-GFP1.8* reporter gene expression between *GMR > aPKC^CA^* and *GMR > aPKC^CA^*; *dRASSF^−/−^* eye discs (Figure 4H,I, quantified in 4J) indicating that Yki target gene expression remains elevated in these eye discs. This result indicates that Yki target gene expression remains elevated when *dRASSF* function is depleted in *GMR > aPKC^CA^* eye discs. This supports the observation that tissue growth was not reduced in *GMR > aPKC^CA^*; *dRASSF^−/−^* adult eyes and demonstrates that increased aPKC activity can lead to the activation of Yki target gene expression independently of *dRASSF*.

In mammalian systems classical RASSF proteins, such as RASSF5A/Nore1A, are known effectors of Ras signalling [21,22,23]. In *Drosophila*, loss of *dRASSF* is able to rescue growth defects in Ras mutant tissue [4], suggesting that *dRASSF* may be a negative regulator of Ras signalling. Activated Ras leads to increased phosphorylation of MAPK (ERK) therefore, we isolated protein extracts from larval eye discs mutant for *dRASSF* alone, expressing *GMR > aPKC^CA^*, or *GMR > aPKC^CA^*; *dRASSF^−/−^* tissue and determined the ratio of pERK *versus* total ERK levels by western blot analysis. As shown in Figure 4F, and quantified in Figure 4F', although a two fold increase in the relative ratio of pERK/ERK was detected in *GMR > aPKC^CA^* discs compared to control wildtype eye discs, the removal of *dRASSF* had no effect on the ratio of pERK/ERK. This result demonstrates that MAPK signalling is not changed in *dRASSF^−/−^* or *GMR > aPKC^CA^*; *dRASSF^−/−^* tissue.

## 4. Discussion and Conclusions

**Figure 5:**
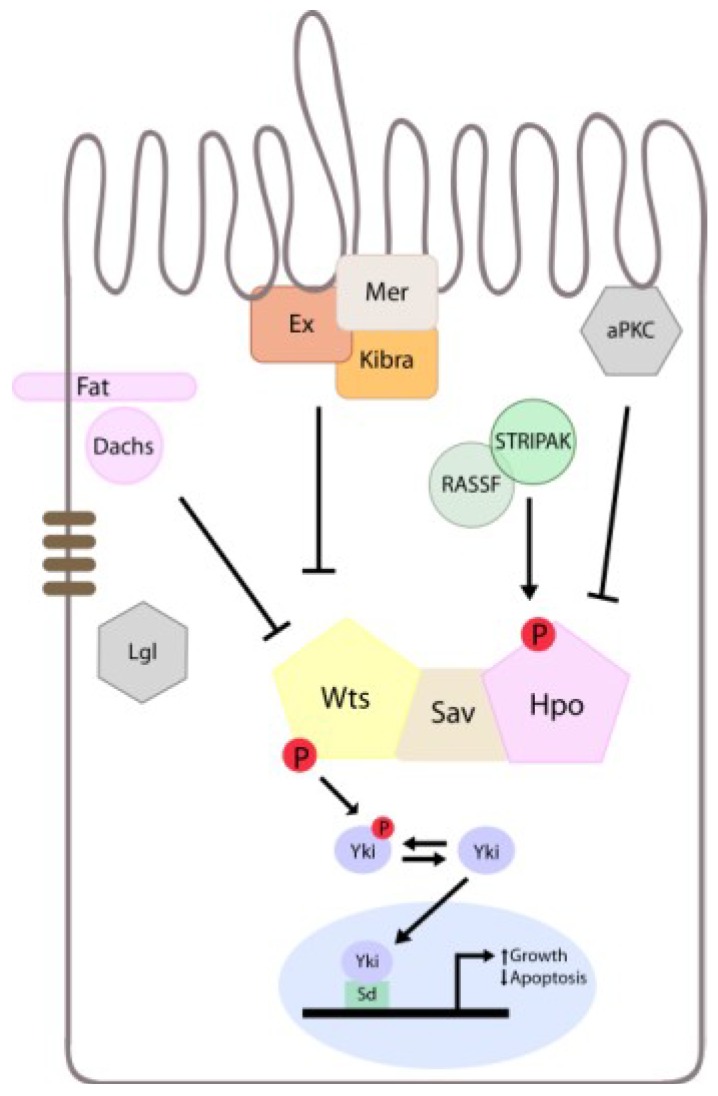
Schematic model of Lgl/aPKC function in Hippo signalling. For simplicity, only proteins relevant to the current study are illustrated in the diagram. Interactions between different upstream components of the Hpo pathway and Yki are shown (positive interactions are illustrated by arrows, inhibitory interactions are depicted with blocked lines). The Hpo pathway controls tissue growth by regulating the subcellular localization of Yki, which when phosphorylated is cytoplasmic and unphosphorylated is nuclear. Yki associates with cognate transcription factors to promote cell proliferation and inhibit apoptosis. Lgl/aPKC regulate Hpo signalling independently of Fat/Dachs, Kibra/Expanded/Merlin and dRASSF/dSTRIPAK complexes.

Here we demonstrate that the apico-basal cell polarity regulators Lgl/aPKC regulate the Hpo pathway independently of upstream inputs Fat/Dachs and the Kibra/Ex/Mer complex. We also show that in addition to Hpo and dRASSF, members of the dSTRIPAK complex Cka and Mob4 are mislocalized in *lgl^−/−^* tissue. We predicted that comislocalization of Hpo and dRASSF observed in *lgl^−/−^* tissue, or eye discs overexpressing *aPKC^CA^*, was critical for deregulation of the Hpo pathway and upregulation of Yki target genes such as *diap1* and *cycE*. However, we found that *dRASSF* was not required for Hpo mislocalization. Surprisingly, removal of *dRASSF* in *lgl^−/−^* tissue, or eye discs overexpressing *aPKC^CA^*, did not suppress tissue overgrowth phenotypes, or reduce *diap1* levels as predicted, but resulted in increased tissue growth. Moreover, knockdown of dSTRIPAK components did not rescue the eye overgrowth defects due to overexpression of *aPKC^CA^*. These data suggest that dRASSF or dSTRIPAK are not required for inhibition of Hpo by aPKC upregulation, and therefore Lgl/aPKC regulates the Hpo pathway by a novel mechanism (Figure 5).

Previous studies in *Drosophila*, have proposed that dRASSF antagonizes Hpo pathway activity by directly competing with Sav for binding to the SARAH domain within Hpo [4]. In addition, dRASSF forms a multimeric protein complex with Hpo and a phosphatase complex (dSTRIPAK) that dephoporylates Hpo, thereby reducing Hpo activity, and promoting Yki target gene expression [5]. We expected that the comislocalization of Hpo and dRASSF, in *lgl^−/−^* tissue, could account for the decreased Hpo activity and increased Yki target gene expression observed [7]. However, in this study, we show that in the developing eye disc, *dRASSF* is not required to mislocalize Hpo in *lgl* mutant tissue. Furthermore, removal of *dRASSF* in *GMR > aPKC^CA^* eye discs failed to reduce elevated levels of *diap1* gene expression. These data indicate that the removal of *dRASSF* in *lgl^−/−^* or *GMR > aPKC^CA^* tissue does not simply lead to the reestablishment of Hpo/Sav protein complexes and subsequent inhibition of Yki target gene expression.

In this study we also demonstrated that the regulatory subunits of the dSTRIPAK complex, Cka and Mob4 colocalized with Hpo, and similar to Hpo, accumulated apically and Cka was mislocalized basolaterally in *lgl^−/−^* tissue. Since dSTRIPAK is a negative regulator of Hpo, its accumulation with Hpo in *lgl^−/−^* tissue might account for the decrease in Hpo activity. However, our data showing that depletion of members of the dSTRIPAK complex did not suppress the overgrown eye phenotype of *GMR > aPKC^CA^*, argues against this. This suggests that Lgl/aPKC regulate Hpo activity by a novel mechanism, although it remains formally possible that dRASSF and dSTRIPAK act redundantly to inactivate Hpo, and that removal of both will be necessary to restore Hpo activity upon *lgl* depletion/*aPKC^CA^* overexpression. Thus, the effect of Lgl/aPKC on the biochemical and functional interactions between dRASSF/dSTRIPAK/Hpo and Hpo/Sav protein complexes warrants further investigation.

We observed a mild increase in tissue growth in *lgl*; *dRASSF^−/−^* and *GMR > aPKC^CA^*; *dRASSF^−/−^* adult eyes suggesting that *dRASSF* acts as a tumor suppressor when Lgl/aPKC activity is altered. Our data, showing that *diap1-GFP1.8* levels are not further elevated in *GMR > aPKC^CA^*; *dRASSF^−/−^* compared to *GMR > aPKC^CA^* eye discs, suggests that increased tissue growth is not due to further inactivation of the Hpo signalling pathway and upregulation of Yki target genes. This raises the possibility that dRASSF regulates additional growth signalling pathways. Consistent with previous data in mammalian and zebrafish systems, we observed an increase in MAPK signalling when the activity of aPKC cell polarity complexes was altered [24,25,26]. Although dRASSF has been previously linked to negative regulation of the Ras signalling pathway, we did not observe any change in pERK levels in *dRASSF* or *GMR > aPKC^CA^*; *dRASSF^−/−^* tissue relative to *GMR > aPKC^CA^*, demonstrating that MAPK signalling is not altered by removal of *dRASSF.* Taken together our data clearly demonstrate that the molecular mechanisms utilized by dRASSF to regulate tissue growth, and the cellular contexts in which they are employed remain to be elucidated. The further exploration of the role of *dRASSF* in *Drosophila* development, and cancer models, should provide exciting insights into the complexities of cancer biology.

## References

[1] Pan D. (2010). The hippo signalling pathway in development and cancer. Dev. Cell.

[2] Richter A.M., Pfeifer G.P., Dammann R.H. (2009). The RASSF proteins in cancer; from epigenetic silencing to functional characterization. Biochim. Biophys. Acta.

[3] Serwood V., Recino A., Jeffries A., Ward A., Chalmers A.D. (2010). The N-terminal RASSF family: A new group of Ras-association-domain-containing proteins, with emerging links to cancer formation. Biochem. J..

[4] Polesello C., Huelsmann S., Brown N.H., Tapon N. (2006). The *Drosophila* RASSF homolog antagonizes the hippo pathway. Curr. Biol..

[5] Ribeiro P.S., Jouse F., Wepf A., Wehr M.C., Rinner O., Kelly G., Tapon N., Gstaifer M. (2010). Combined functional genomic and proteomic approaches identify a PP2A complex as a negative regulator of Hippo signalling. Mol. Cell.

[6] Gateff E., Schneiderman H.A. (1974). Developmental Capacities of Benign and Malignant Neoplasms of *Drosophila*. Wilhelm Roux’ Arch. Entwicklungsmech. Org..

[7] Grzeschik N.A., Parsons L.M., Allott M.L., Harvey K.F., Richardson H.E. (2010). Lgl, aPKC, and Crumbs regulate the Salvador/Warts/Hippo pathway through two distinct mechanisms. Curr. Biol..

[8] Lee T.V., Luo L. (2001). Mosiac analysis with a repressible cell marker (MARCM) for *Drosophila* neural development. Trends Neurosci..

[9] Bennett F.C., Harvey K.F. (2006). Fat cadherin modulates organ size in *Drosophila* via the Salvador/Warts/Hippo signalling pathway. Curr. Biol..

[10] Silva E., Tsatskis Y., Gardano L., Tapon N., McNeill H. (2006). The tumor-suppressor gene fat controls tissue growth upstream of expanded in the hippo signalling pathway. Curr. Biol..

[11] Willecke M., Hamaratoglu F., Kango-Singh M., Udan R., Chen C., Tao C., Zhang X., Halder G. (2006). The fat cadherin acts through the hippo tumor-suppressor pathway to regulate tissue size. Curr. Biol..

[12] Hamaratoglu F., Willecke M., Kango-Singh M., Nolo R., Hyun E., Tao C., Jafar-Nejad H., Halder G. (2006). The tumour-suppressor genes NF2/Merlin and Expanded act through Hippo signalling to regulate cell proliferation and apoptosis. Nat. Cell Biol..

[13] Cho E., Feng Y., Rauskolb C., Maitra S., Fehon R., Irvine K.D. (2006). Delineation of a Fat tumor suppressor pathway. Nat. Genet..

[14] Mao Y., Rauskolb C., Cho E., Hu W.L., Hayter H., Minihan G., Katz F.N., Irvine K.D. (2006). Dachs: An unconventional myosin that functions downstream of Fat to regulate growth, affinity and gene expression in *Drosophila*. Development.

[15] Baumgartner R., Poernbacher I., Buser N., Hafen E., Stocker H. (2010). The WW Domain Protein Kibra Acts Upstream of Hippo in *Drosophila*. Dev. Cell.

[16] Genevet A., Wehr M.C., Brian R., Thompson B.J., Tapon N. (2010). Kibra is a regulator of the the Salvador/Warts/Hioo Signalling Network. Dev. Cell.

[17] Yu J., Zheng Y., Dong J., Klusza S., Deng W.U., Pan D.J. (2010). Kibra Functions as a Tumor Suppressor Protein that Regulates Hippo Signalling in Conjunction with Merlin and Expanded. Dev. Cell.

[18] Büther K., Plaas C., Barnekow A., Kremerskothen J. (2004). KIBRA is a novel substrate for protein kinase C zeta. Biochem. Biophys. Res. Commun..

[19] Goudreault M., D’Ambrosi L.M., Kean M.J., Mullin M.J., Larsen B.J., Sanchez A., Chaudhry S., Chen G.I., Sicheri F., Nesvizhskii A.I. (2009). A PP2A phosphatase high density interaction network identifies a novel striatin-interacting phosphatase and kinase complex linked to the cerebral cavernous malformation 3 (CCM3) protein. Mol. Cell. Proteomics.

[20] Wu S., Liu Y., Zheng Y., Dong J., Pan D. (2008). The TEAD/TEF family protein Scalloped mediates transcriptional output of the Hippo growth-regulatory pathway. Dev. Cell.

[21] Vavvas D., Li X., Avruch J., Zhang X.F. (1998). Identification of Nore1 as a potential Ras effector. J. Biol. Chem..

[22] Vos M.D., Ellis C.A., Elam C., Iku A.S.U., Taylor B.J., Clark G.J. (2003). RASSF2 Is a Novel K-Ras-specific Effector and Potential Tumor Suppressor. J. Biol. Chem..

[23] Vos M.D., Martinez A., Ellis C.A., Vallecorsa T., Clark G.J. (2003). The Pro-apoptotic Ras Effector Nore1 May Serve as a Ras-regulated Tumor Suppressor in the Lung. J. Biol. Chem..

[24] Castoria G., Migliaccio A., di Domenico M., Lombardi M., de Falco A., Varricchio L., Bilancio A., Barone M.V., Auricchio F. (2004). Role of atypical protein kinase C in estradiol-triggered G1/S progression of MCF-7 cells. Mol. Cell. Biol..

[25] Nolan M.E., Aranda V., Lee S., Lakshmi B., Basu S., Allred D.C., Muthuswamy S.K. (2008). The polarity protein Par6 induces cell proliferation and is overexpressed in breast cancer. Cancer Res..

[26] Reischauer S., Levesque M.P., Nüsslein-Volhard C., Sonawane M. (2009). *Lgl2* executes its function as a tumor suppressor by regulating ErbB signalling in the *zebrafish* epidermis. PLoS Genet..

